# Professionalism, Leadership, and Spiritual Awareness: Reflections from Four Decades in Plastic Surgery

**DOI:** 10.1055/s-0046-1822795

**Published:** 2026-06-23

**Authors:** Rajeev B. Ahuja

**Affiliations:** 1 Department of Plastic Surgery, Sir Gangaram Hospital, New Delhi, India

कर्तव्ये न विचाल्यन्ते, सत्ये धृतिमुपाश्रिताः।

नेतारः स्युः विनीताश्च, धर्मे युक्ताः सदाऽनघाः॥ Anonymous

Translation: “Those steadfast in duty and anchored in truth become leaders who act with humility and righteousness.”

## Introduction

These words, written more than two millennia ago, resonate strongly even today. In my over four decades in the practice of plastic surgery, I have witnessed profound changes—technological revolutions, evolving health care systems, and shifting expectations of the medical profession and the patients. Yet, despite these changes, the enduring principles and interconnected pillars that continue to form the bedrock of a meaningful career are professionalism, leadership, and spiritual awareness. These elements are not learned solely from textbooks or formal training programs. Rather, they are cultivated gradually through years of patient care, teaching, mentorship, and reflection.

The ancient surgical text, the Sushruta Samhita, 1 also reminds us that the ideal physician must combine knowledge, technical skill, courage, and compassion.

शास्त्रं दृढं च हस्तस्य, शुद्धिः कर्मणि नित्यदा।

धैर्यं दया च वैद्यस्य, सिद्धेः कारणमुच्यते॥

Translation: “Firm knowledge of the science, steady hands in practice, purity in conduct, courage, and compassion—these are said to be the causes of a physician's success.”

## Professionalism: The Ethical Compass

In the early years of surgical training, emphasis is placed on mastering technique and acquiring knowledge. As young surgeons, we often equate professional success with surgical skill and academic recognition. While these are undoubtedly important, experience teaches that technical competence alone is insufficient to sustain a meaningful and satisfying career in medicine.

Professionalism, in its truest sense, represents a lifelong commitment to integrity, accountability, and the pursuit of patients' trust. Patients are hardly ever concerned with the intricacies of a surgical procedure, but they remember the compassion, honesty, and reassurance offered by their doctor. Herbert M. Swick 2 previously stated that professionalism encompasses the behaviors through which physicians demonstrate their commitment to patients and society. In practice, this means maintaining ethical standards, accepting responsibility for outcomes, and continually striving to improve. There's a commitment to lifelong learning. My insights into ethical practices in plastic surgery, while delivering evidence-based treatment, grew even more when I had to read the literature to write a review article on the topic. 3

As stated by Sridhar, 4 when I too began my career, many of the technologies and techniques we now consider routine did not exist. Not that there was any shortage of text on the techniques used in reconstruction. Converse 5 alone had 4,000 pages, which I bought for $375 in 1982, quite contrary to the majority of Gen Z and M, who pride themselves on reading books on their iPhones or iPads. The willingness to adapt and continue learning has been essential to remaining relevant in an ever-changing field. In fact, in my Gilles' Oration in 2007, I stated that almost 80% of the techniques that I practiced then were relearned in the years after my M.Ch. in 1983. Prof. C. P. Sawhney had once remarked to me that everything couldn't be taught in a two-year M.Ch. program, and that we had to teach and self-learn over the subsequent years.

**Fig. 1:**
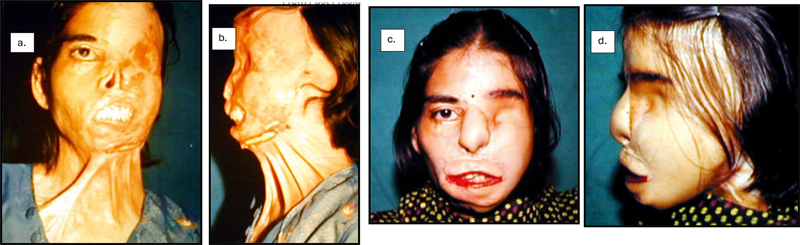
( **A** and **B** ) Preoperative views. ( **C** and **D** ) Postoperative views after almost 2 years of reconstructive procedures involving split-skin graft after the release of neck contracture, extracorporeal radial artery forearm flap-extended to above the cubital fossa by a delay procedure, nose reconstruction with an arm tube-flap, release of upper and lower lip ectropion and full-thickness skin graft, and eyebrow reconstruction with a composite graft from the scalp.

Surgery is a discipline that constantly reminds us of unexpected challenges and complications, as well as our limitations. A professional accepts these moments with honesty, learns from them, and reflects with humility. I remember an extremely challenging case for post-burn reconstruction in the early nineties. A young epileptic female fell on a “Chullah” and completely burnt her face, and she lost her left eye. Long after her burns had healed, she presented with a most grotesque deformity of face and neck. She presented a challenge, and through multiple stages of reconstruction, this extremely poor patient could be provided with a semblance of a face to return to society ( Fig. 1 ). This kind of unique satisfaction is what we should all yearn for, beyond our material and professional growth.

## Leadership: Beyond the Operating Room

As one progresses in a medical (surgical) career, the role and responsibilities inevitably evolve and extend beyond direct patient care to include teaching, mentoring, and guiding teams. In this context, leadership becomes an integral part of professional life.

This leadership is not defined by titles or administrative positions. Instead, it emerges through influence, example, and the ability to inspire others. A senior surgeon is often looked upon as a guide by younger colleagues and residents. The way we conduct ourselves—in the operating theater, during ward rounds, in moments of crisis, or even socially—shapes the professional culture of the team around us.

Effective leadership also requires emotional intelligence, a concept popularized by Daniel Goleman. 6 The surgical environment can be intense and demanding, and the ability to remain composed, communicate clearly, and understand the emotions of others becomes crucial. Teams function best when they feel respected and supported, and they can appreciate your contribution to their professional growth.

One of the most rewarding aspects of a long surgical career has been the opportunity to mentor younger surgeons. Thus, we were the first to launch the Diplomate of National Board (DNB) program in Plastic Surgery in 1997 when I assumed the charge of the head of the department at Lok Nayak Hospital and Maulana Azad Medical College. As Secretary of APSI (Association of Plastic Surgeons of India), we tasked a committee to overhaul the M.Ch. curriculum and the same was submitted to the National Board of Examinations to start the DNB program. Watching trainees evolve into confident and compassionate professionals is a privilege that perhaps surpasses even the satisfaction of surgical success. True leadership lies not in personal achievement but in helping others realize their potential, which then becomes your legacy. Today, the country is dotted with bright plastic surgeons who have journeyed through our department and made us proud.

Your colleagues and seniors notice you when you push the boundary. You present good academic work, you have meaningful publications, and you volunteer sincerely to help in the growth of your department, institution, or your professional society. Such traits get you elected to offices in professional associations nationally, and some may even get catapulted to horizons beyond.

## Reflections from a Personal Journey in Plastic Surgery

The following relatable anecdotes mark a journey peppered with drive, struggle, and pushing boundaries.

कर्मण्येवाधिकारस्ते मा फलेषु कदाचन।

मा कर्मफलहेतुर्भूर्मा ते सङ्गोऽस्त्वकर्मणि॥ Bhagavad Gita (Chapter 2, Verse 47),

Translation: “You have the right to perform your prescribed duties, but not to the fruits of those actions. Do not be motivated by rewards, nor have attachment to inaction.”

The most meaningful and memorable two years were the ones spent at Canniesburn Hospital in Glasgow, Royal Marsden Hospital in London, and the Mount Vernon Hospital in Middlesex, from 1986 to 1988 on a Commonwealth Fellowship program to learn microsurgery and head and neck cancer reconstruction. The learning, encouragement, and appreciation imbibed in those years have stood with me in shaping my future career. A few weeks into the Fellowship, I was asked, “Where have you been trained for Plastic Surgery?” My response, “India,” was unbelievable to their ears! A couple of months later, I was asked by my Consultant, Mr. David S. Soutar, if I would like to teach on the “Canniesburn Flap Course?” What a question for a youngster aspiring to join the course and waiting for an opportune time to request a fee exemption! Mr. Soutar also gave us (me and another fellow, Horatio D'Costa from Portugal) the key to the microlaboratory set up by Bob Acland, for practicing on rats. He then went on to recommend extending my Fellowship period by a year and fixed my rotation at the other two hospitals to broaden my experience. The operating room sister in charge gifted me with clogs to carry as a souvenir, which I continue to wear till today while operating. I remain indebted to them.

Mr. Nick M. Breach at the Royal Marsden taught me that doing routine free flaps was actually a “macro” version of “microsurgery” and loupe magnification was sufficient. Consequently, while training with him, I bought three loupes (6 × , 4 × , and 2 × ), along with several microsurgical instruments and a Martin bipolar cautery machine, for use back home on patients at a government hospital.

The nine months at Mount Vernon Hospital, working with Mr. Roy Sanders and Mr. Douglas Harrison, provided further opportunities to learn newer techniques in cleft craft, hypospadias, facial palsy reconstruction, and esthetic surgery.

I had so much love and zest to return to India that even the offer of a Consultant position at Birmingham General Hospital, by Mr. John Gower in 1994 (without having appeared for PLAB [Professional and Linguistic Assessments Board] or FRCS [Fellowship of the Royal College of Surgeons]), on my second fellowship visit to the United Kingdom, couldn't lure me enough to stay back.

The innate desire to publish and present offered several blessed opportunities. During my sabbatical in the United Kingdom, I had the privilege of interacting with young minds across the world, and it honed the manuscript of my first two individual publications in Plastic and Reconstructive Surgery (PRS) on the rotation template flap, which I had designed, and employed the procedure on several patients just before leaving India. 7 8 This ultimately took over two decades to be validated and led to an invitation to contribute chapters in textbooks. 9 10

I first participated in the IPRAS (International Confederation for Plastic, Reconstructive and Aesthetic Surgery) Conference in 1999 in San Francisco. Those were nondigital days, and Kodak projectors with carousels were in vogue. I had to buy two projectors to practice simultaneous slide projections, and precisely time the presentations to eight minutes, with my eight-year-old daughter being the projectionist and the timekeeper. This was the zeal to be as flawless as possible, a habit to last a lifetime.

A very interesting coincidence occurred during the Annual Conference of Indian Society for Cleft Lip, Palate and Craniofacial Anomalies (ISCLPCA) in 2005 at Hyderabad, India. I was invited to speak on my technique of “Limited open technique of primary rhinoplasty on cleft lip patients,” and Philip K. T. Chen was invited to speak on his technique of “Semi-open rhinoplasty.” Luckily, I spoke just before him, and both of us were surprised that the techniques were identical. Both of us had even sent it for publication in the same year (2005), and eventually both got published in 2006. He published in a Chapter in Mathes, 11 and mine was in the *Cleft and Craniofacial Journal* . 12 The lesson: Don't keep mulling over your ideas. Execute them and publish as soon as possible, because there are many people thinking of the same idea at the same time.

## Spiritual Awareness: The Inner Dimension of Healing

While professionalism and leadership address the external responsibilities of medical practice, spiritual awareness addresses its internal dimension. Only with spiritual awareness and compassion can you contribute selflessly. Spiritual awareness, in this context, does not necessarily refer to religious belief. Rather, it reflects a broader sense of mindfulness, reflection, and connection with “Karma.” This awareness develops naturally over years of practice.

Burn surgeons frequently encounter terrible suffering, uncertainty, and difficult decisions. Some cases end in triumph; others leave us with lingering questions and reflection. Education is the basic ingredient in delivering good burn care. In India, we have regular teaching programs on burn management through the National Academy of Burns-India, ever since it was established in 1993. But internationally, there were only two recognized courses in burn management: the EMSB course (by the European Burn Association) and the ABLS course (by the American Burn Association), both emphasizing the emergency management of severe burns. David Mackie, the President of the International Society for Burn Injuries (ISBI) from 2010 to 2012, invited me to deliver a keynote address on “Burn Education in LMICs - Seeking Newer Paradigms” at the Edinburgh Congress in 2012. This lecture detailed the need and the outline for a burn course beyond 48 hours, which ISBI then adopted. This course, intended for professionals from low- and middle-income countries (LMICs), is attended more enthusiastically by people from the developed world.

As we all know, the problem of burns is “endemic” in LMICs, especially the Indian subcontinent. I was acutely aware of the lack of a standard “Guidelines for Burn Management” in a low-resource environment. During my term as the President of ISBI from 2014 to 2016, I, along with Mike Peck (USA), grouped well-known burn surgeons across the world to frame the ISBI Practice Guidelines for Burn Care, 13 14 which, for the first time, Elsevier made freely downloadable on our lobbying.

The dilemma of having to treat with limited resources despite the ability to deliver better care can be a daunting emotional burden. God alone knows how many occasions I have intensely prayed for the success of a complicated surgery. Consistent success not only requires intellectual ability and technical skill but also “the hand of God.” Long waiting lists of patients with post-burn disability were dismaying. After completing my term as the President of ISBI, I vowed to establish a foundation to help such patients from underprivileged strata. Thus, the Burn Healing Foundation (burnhealingfoundation.com) was formed in 2020, in the era of coronavirus disease lockdown.

## Conclusion

Looking back over four decades in plastic surgery, it becomes clear that the most meaningful achievements are rarely the ones measured in academic titles or surgical numbers. Instead, they lie in the trust earned from patients, the growth of students and trainees, respect from colleagues, and the knowledge that one has contributed in whatever way possible, however modestly.

Practices such as mindfulness, contemplation, or simple introspection can help one maintain emotional balance and avoid burnout. They remind us that medicine is not merely a profession but a calling that involves both scientific skill, human compassion, and the “will of God.”

Medicine continues to evolve at an extraordinary pace, yet the essential values that define the profession remain constant. For young doctors embarking on their careers, technical skill and academic achievement will always be important. However, true fulfillment in medicine arises from a broader perspective—one that integrates professional excellence, responsible leadership, and a deeper sense of purpose. When nurtured together, they allow physicians not only to heal patients but also to inspire colleagues, guide future generations, and preserve the humanistic spirit of medicine.
